# A collection of European sweet cherry phenology data for assessing climate change

**DOI:** 10.1038/sdata.2016.108

**Published:** 2016-12-06

**Authors:** Bénédicte Wenden, José Antonio Campoy, Julien Lecourt, Gregorio López Ortega, Michael Blanke, Sanja Radičević, Elisabeth Schüller, Andreas Spornberger, Danilo Christen, Hugo Magein, Daniela Giovannini, Carlos Campillo, Svetoslav Malchev, José Miguel Peris, Mekjell Meland, Rolf Stehr, Gérard Charlot, José Quero-García

**Affiliations:** 1 UMR 1332 BFP, INRA, Univ. Bordeaux, Villenave d’Ornon 33140, France; 2 NIAB EMR, East Malling, ME19 6BJ, UK; 3 IMIDA, Murcia Institute of Agri-Food Research and Development, Murcia 30150, Spain; 4 INRES—Horticultural Science, University of Bonn, 53121 Bonn, Germany; 5 Fruit Research Institute (FRI), Čačak 32000, Republic of Serbia; 6 University of Natural Resources and Life Sciences, Vienna, Department of Crop Sciences, Division of Viticulture and Pomology, Vienna 1180, Austria; 7 Agroscope Changins—Wädenswil Research Station ACW, 1964 Conthey, Switzerland; 8 Walloon Agricultural Research Center (CRA-W), Gembloux 5030, Belgium; 9 CREA, Council for Agricultural Research and Economics, Fruit Tree Research Unit of Forlì, Forlì 47121, Italy; 10 Scientific and Technological Research Center of Extremadura (CICYTEX), Guadajira (Badajoz) 06187, Spain; 11 Fruit Growing Institute—Plovdiv, Plovdiv 4004, Bulgaria; 12 IRTA, Experimental Station of Lleida, Lleida 25003, Spain; 13 Norwegian Institute of Bioeconomy Research—NIBIO Ullensvang, Lofthus N-5781, Norway; 14 Fruit Research Center Jork, Jork D-21635, Germany; 15 Ctifl, 751 chemin de Balandran, Bellegarde 30127, France

**Keywords:** Plant sciences, Climate change, Phenology

## Abstract

Professional and scientific networks built around the production of sweet cherry (*Prunus avium* L.) led to the collection of phenology data for a wide range of cultivars grown in experimental sites characterized by highly contrasted climatic conditions. We present a dataset of flowering and maturity dates, recorded each year for one tree when available, or the average of several trees for each cultivar, over a period of 37 years (1978–2015). Such a dataset is extremely valuable for characterizing the phenological response to climate change, and the plasticity of the different cultivars’ behaviour under different environmental conditions. In addition, this dataset will support the development of predictive models for sweet cherry phenology exploitable at the continental scale, and will help anticipate breeding strategies in order to maintain and improve sweet cherry production in Europe.

## Background & Summary

The impact of climate change on plant phenology has been described in recent decades, highlighting a hastening of flowering phenology in response to increasing winter and spring temperatures^1–7^. Long records of flowering dates have been proven extremely valuable to reconstruct past phenology and to predict phenology under future climatic scenarios. In Japan for example, flowering dates of cherry trees (*Prunus jamasakura*) have been recorded for centuries and analyses of these records have revealed that cherry flowering is currently occurring earlier than at any time in the previous seven to 12 centuries, due to the impact of warming and urbanization on phenology^5,8,9^. This earlier flowering trend has been observed for other fruit trees^10–14^, which are particularly vulnerable to temperature changes due to their long life span. Peach (*Prunus persica*), apricot (*Prunus armeniaca*), almond (*Prunus dulcis*), plum (*Prunus salicina* and *domestica*) and sweet cherry (*Prunus avium* L.) are amongst the most commercially important *Prunus* fruit tree species planted in temperate climate zones. In Europe, cherry tree blossom of early maturing cultivars showed an advance up to 4.7 days/°C in Germany^10,14^, and warmer winters have dramatically affected the sweet cherry production in South-Western France, with a 30% yield in 2007. Warmer winters can as well be associated with delayed spring phenology for some species and, occasionally resulting in abnormal flowering phenology and reduced productivity^15–18^. Models for chill availability predict an increase in the delaying effect of mild winters as temperature increase becomes more pronounced^6,19,20^, especially in warmer locations^21^. In the context of substantial changes to environmental conditions induced by climate change, it will be essential that plant cultivars are well adapted to warmer winter and spring temperatures and to more extreme climatic events such as erratic spring frosts and summer heat waves. This is especially true for perennial fruit crops, which require more than a decade before a new cultivar is released.

Large phenological datasets are key for the development of phenological models (e.g., refs 22–25), which are valuable tools to support breeding strategies. Although recent studies have shown the value of a wide range of data^26,27^, most analyses for fruit tree crops are based on phenological data for a very limited number of experimental sites, and rarely include more than two cultivars within a species (e.g., refs 28,29).

Sweet cherry trees are particularly interesting for phenology studies, their long orchard life providing the potential for long-term datasets. Reference cultivars have been planted and observed for decades for phenology and productivity traits in trials dedicated to new hybrids characterization. For example, at the Fruit Experimental Station (Toulenne, INRA Bordeaux, France), phenological data have been recorded for ‘Burlat’ cultivar for 35 years. Consequently, large phenological datasets are available for reference cultivars in many European orchards involved in breeding programmes. Despite this, long historical datasets of fruit tree phenology are rarely analysed together or made available to the scientific community. A few analyses on sweet cherry phenology in Europe were published using the phenological observations of fruit trees by the German Weather Service (DWD)^10,12^, from non-publicly available datasets^30,31^ or from the PEP725 data^14^. In addition, published studies often focus on specific location^10,12,30–33^.

**Figure 1:**
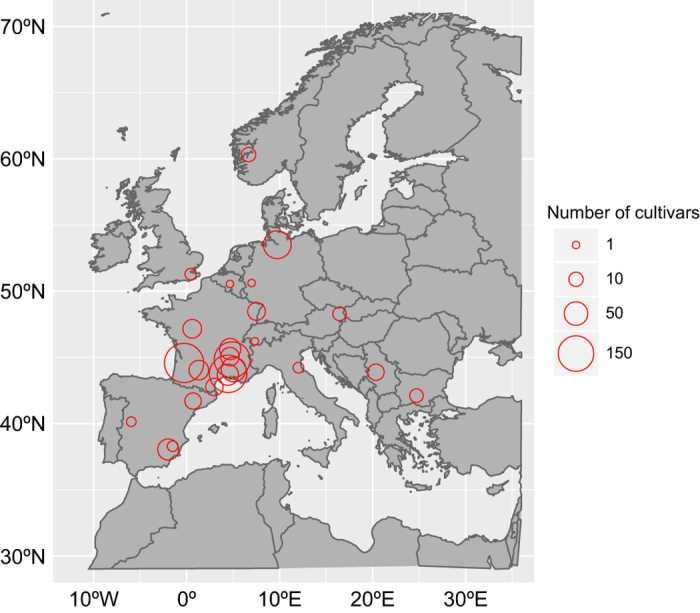
Experimental orchards location. Flowering and maturity dates were recorded in 25 sites from 11 European countries, indicated by the red circles. Size of the circle is proportional to the number of cultivars recorded in each site.

In this study, we describe a unique dataset of sweet cherry flowering and maturity records for 25 sites in Europe (Fig. 1) with highly contrasted climates. Past studies showed that phenology data spanning 20 or 30 years were valuable for climate change related analyses^7,34^. Thus the dataset presented here, with an overall 37 year-period (1978–2015, Fig. 2) will be valuable for phenology and climate change studies. This dataset covers a wide range of European latitudes and longitudes and is unique in its collection of cultivars (between 1 and 191 cultivars per site), each cultivar being represented by clones of the same original tree in each country, which supports robust analyses of plasticity and response to climatic conditions.

**Figure 2:**
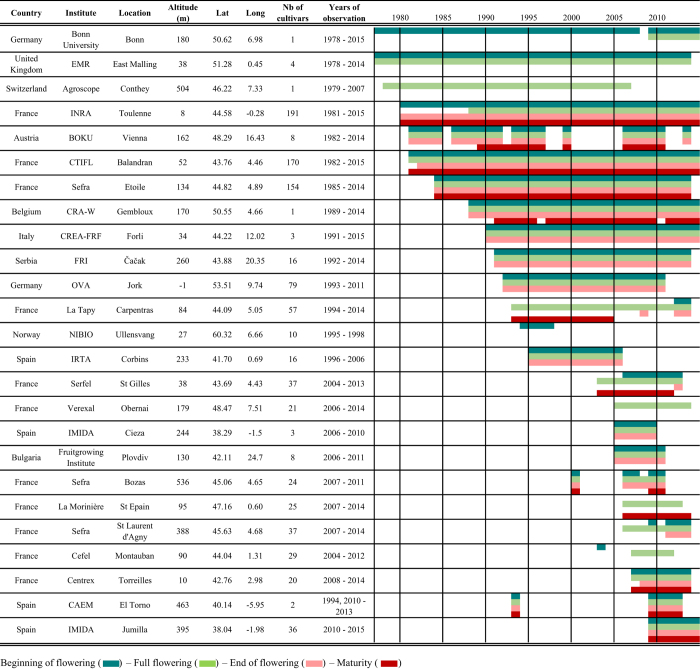
Description of data source, time-span, number of observed cultivars, and location within Europe.

**Figure 3:**
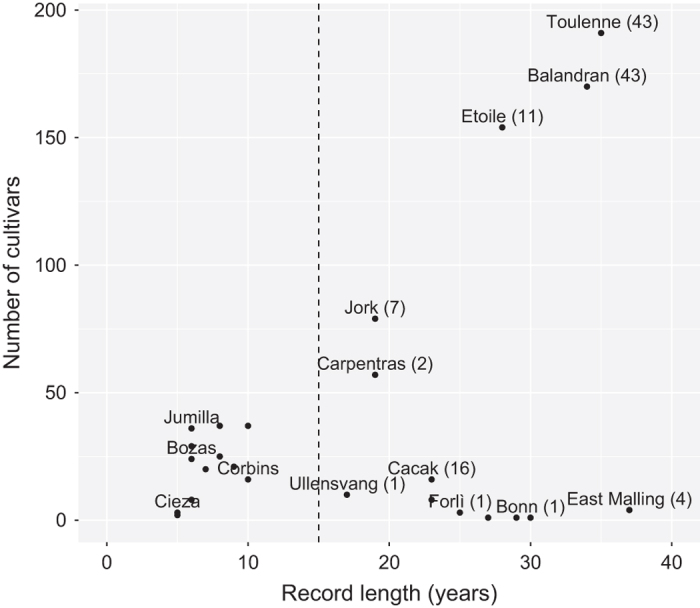
Information on the number of observed cultivars and the overall record length for each site. Some site names were omitted the ease the comprehension. The numbers between brackets indicate the number of cultivars with more than 15 years of observation. The dash line indicate the 15 years limit for the record length, criteria mentioned in (Fu *et al.* ^7^) as sufficient to perform climate change response analysis.

Since data were collected from various experimental stations, the dataset is not homogeneous regarding the number of cultivars (Figs 1 and 2, Table 1) or the record length (Fig. 2). Past research have shown the value of using heterogeneous records combined from different sites and cultivars for the evaluation of climate change response and phenology modelling approaches (e.g., refs 21,35–38). In particular, phenology models have been successfully tested and optimized using data sourced from different sites^27,38^. Therefore, we want to highlight the value of this dataset, combining data from various geographical sites and contrasted cultivars, for potential multi-environment analyses yet to be implemented. 13 out of 25 sites meet the criteria of more than 15 recorded years that was shown to be useful for climate change analysis^7^ (Fig. 3; Table 1). Single site analyses can thus be applied to track phenological climate shifts in a given environment. Subsequently, phenology models can be further evaluated by pooling data across geographically and climatically varied sites, leading to a better knowledge of climate-phenology relations. Extreme European climate, e.g., South of Spain, can be used to investigate climate analogues for projected climatic scenarios^39^. In addition, for eight sites, two or more cultivars were observed for at least 15 years (Fig. 3). Between cultivar differences can be assessed at a single site and across sites with common cultivars. These analyses can reveal whether some cultivars are more susceptible to evolution in climatic conditions. The dataset offers the possibility to study different flowering phase data, namely beginning, full and end of flowering, together with maturity dates for some cultivars (Table 1), and to perform sequential phenology phase assessments.

Overall, despite the fact that they are heterogeneous, data from the different sites can be combined in integrated analysis to study their responses to environment. Such dataset is extremely valuable for characterizing the phenology response to climate change and the plasticity of the different cultivar behaviour under various environmental conditions. In addition, these records can support the development of predictive models exploitable at the continental scale that can be used by growers and breeders, and to anticipate breeding strategies in order to maintain and improve sweet cherry production in Europe.

## Methods

**Figure 4:**
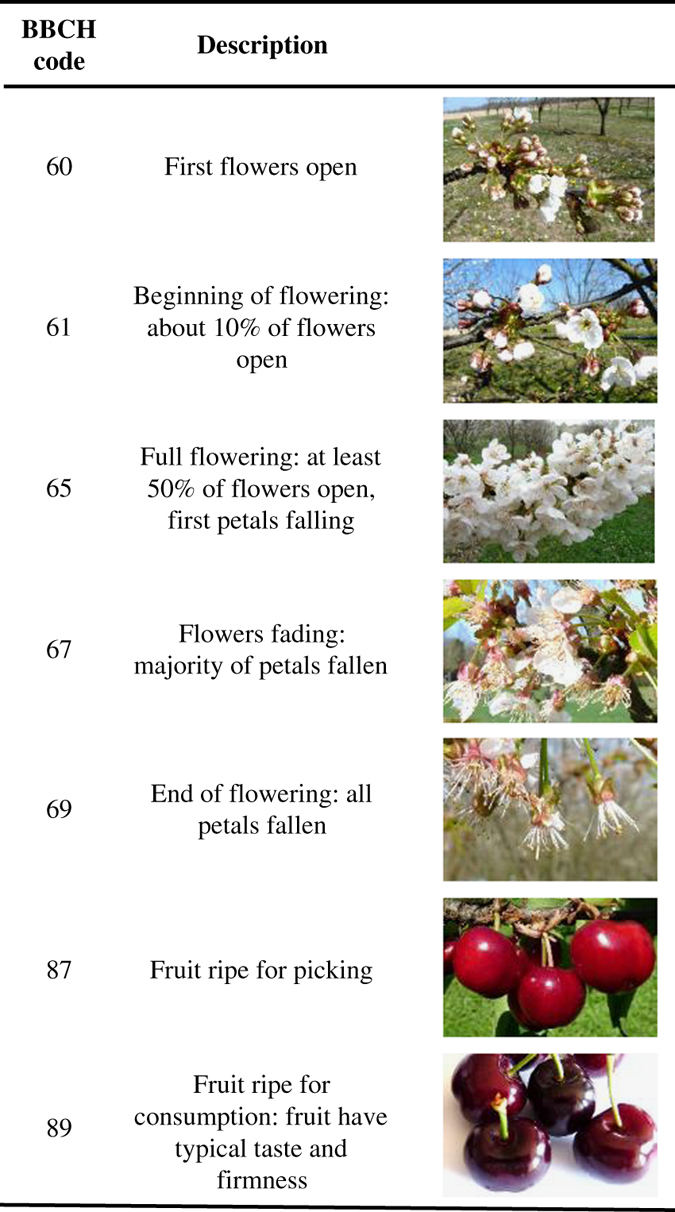
BBCH descriptors (Meier^40^) corresponding to the flowering and maturity stages observed in the dataset. Pictures @INRA.

Sweet cherry phenological data were collated from French and European networks. Established in 1952, CTIFL is a non-profit organization involved in the French fruit and vegetable industry. It developed a private database dedicated to information on cultivars planted in experimental orchards. Flowering and maturity dates for up to 191 reference cultivars grown in French experimental stations were extracted from the database. At the European scale, in the context of the COST Action 1104 (2012–2016; https://www.bordeaux.inra.fr/cherry/), which aimed at creating a dynamic network of scientists and other professionals conducting research to improve sweet or sour cherry production in Europe, we established a working group (WG) for phenology studies. Flowering and maturity dates together with the protocol details for the observations were collected. Although a standardisation of the recorded stages is on-going within the group, past observation standards for the different flowering stages are not homogeneous and are described in Fig. 4 and Table 2. They correspond to a percentage of open flowers or fallen petals. Since the development of the BBCH scale (*Biologische Bundesanstalt, Bundessortenamt und Chemische Industrie*), this standard has been applied as a coding system for the characterization of the entire developmental cycle of annual and perennials plants^40,41^. Here, where possible, we associated the recorded stages, as defined in each experimental location for observations, with the corresponding BBCH stage (Table 2). In every location, one or two observers were in charge of recording the phenology dates. At the end of the season, records were added to the dataset. We calculated the length of the flowering season, which is the number of days between beginning and end of flowering, where these dates were available.

## Data Records

Flowering and maturity dates from all sites can be found in the dataset file stored in the Dryad Digital Repository (Data Citation 1). The spread sheet consists of a table with the description of all phenological data (Table 3). The experimental sites are described by name, latitude, longitude and altitude. Each row corresponds to the dates (beginning of flowering, full flowering, end of flowering, beginning of maturity) documented each year for one tree when available, or the average of several trees for each cultivar. For registration reasons, one cultivar can be registered and observed under different clone numbers, ranging from 1 to 7 clone accession numbers, so the clone number was indicated when available. The cultivar name was always indicated and when available the rootstock information was provided. Dates recorded were also provided as day of year (starting with 1 for January 1st) and the duration of flowering was calculated (days).

## Technical Validation

All data were checked for consistency and anomalous values were corrected or removed (Supplementary Table 1). Some old cultivars, that can be found in many countries, have names that differ slightly between the sites so we arbitrarily chose one common name: ‘Badacsony’ was selected as the common name for ‘Badacsony’, ‘Badacsoner’ and ‘Badacsonyi’; ‘Francesca’ and ‘Francessca’ were regrouped as ‘Francesca’. In total, 51 records were corrected (Supplementary Table 1).

**Figure 5:**
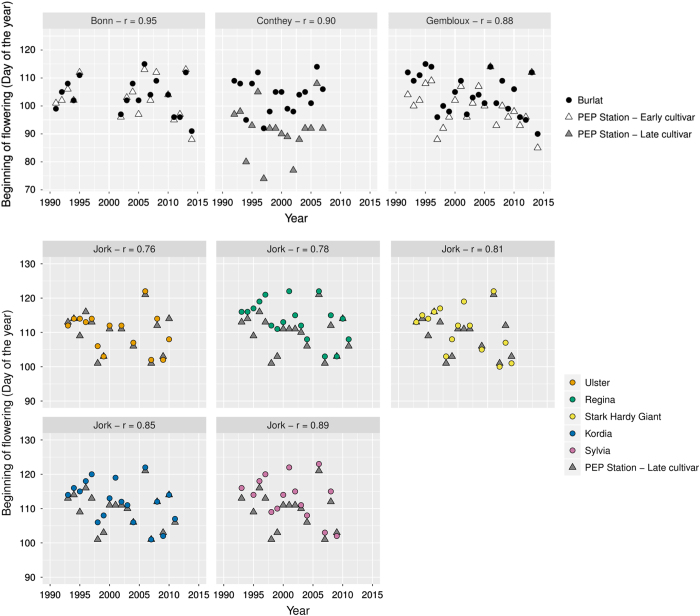
Comparison of flowering dates in days (from 1st of January) for 6 cultivars from our dataset and, unless otherwise stated, for BBCH60 stage for the ‘Early cultivar’ and ‘Late cultivar’ collected from PEP725 (http://www.pep725.eu) for stations geographically close to our experimental sites. For the Bonn site, PEP725 data were obtained from the station 1360, located in Zülpich (Germany; longitude: 6.67; latitude: 50.68; distance from Bonn: 23 km). Gembloux data were compared to flowering dates from the station 1498, located in Übach-Palenberg (Germany, longitude: 6.12; latitude: 50.92; distance: 110 km). For Jork, PEP725 data were obtained from the station 346, located in Hamburg-Altengamme (Germany; longitude: 10.28; latitude: 53.43; distance: 37 km). Conthey data were compared to full flowering (BBCH stage 65) dates from station 3260 (Müllheim, Germany; longitude: 7.63; latitude: 47.82, distance: 178 km). Spearman’s correlation coefficient was calculated for each site between our data and corresponding PEP725 data.

For records of more than 15 years, we checked the consistency of collected data between sites. This cross-checking showed data for a given cultivar were highly correlated, even for sites as far as 400 km from each other (Table 4), confirming that the collected data are consistent. In addition, when possible, we chose to compare our data to similar phenological observational records from the European phenology database PEP725 (http://www.pep725.eu). Data for ‘early cultivar’ and ‘late cultivar’ were retrieved from the database and correlated with close-by sites when at least 15 common years of data were available. We identified PEP stations located within a range of 200 km for Bonn, Conthey, Gembloux and Jork. Strong correlations and minimal differences were found between our data and the flowering dates recorded and validated in PEP725 (Fig. 5). Flowering dates records for six cultivars met the criteria of 15 year-records and the Spearman correlations were all higher then 0.76, regardless of the flowering precocity of the cultivar or the site (Fig. 5).

## Usage Notes

The sweet cherry phenology dataset was collected with the objective to support climate change and phenological analyses for varied European environments. These data can be associated with other *Prunus avium* flowering data provided by the European phenology database PEP725 (http://www.pep725.eu) to perform a wide evaluation of phenology in early and late cultivars.

## Additional Information

**How to cite this article:** Wenden, B. *et al.* A collection of European sweet cherry phenology data for assessing climate change. *Sci. Data* 3:160108 doi: 10.1038/sdata.2016.108 (2016).

**Publisher’s note:** Springer Nature remains neutral with regard to jurisdictional claims in published maps and institutional affiliations.

## Supplementary Material



Supplementary Table 1

## Figures and Tables

**Table 1:** Overall description of the data records.

**Site**	**Number of observed cultivars**	**Number of data points**				
		**Beginning of flowering**	**Full flowering**	**End of flowering**	**Maturity**	**Total**
Balandran	170	2530	2714	2192	2555	9991
Bonn	1	30	6			36
Bozas	24	72	100	87	32	291
Čačak	16	338	336	286	49	1009
Carpentras	57		552	8	282	842
Cieza	3	15	15	15		45
Conthey	1		29			29
Corbins	16	157	156	147	10	470
East Malling	4	167	167	167		501
El Torno	2	10	10	9	10	39
Etoile	154	1675	1686	1409	1229	5999
Forli	3	38	38	31	7	114
Gembloux	1	27	27	27	22	103
Jork	79	685	685	682		2052
Jumilla	36	234	234	205	234	907
Montauban	29	9	79			88
Obernai	21		154		95	249
Plovdiv	8	45	45	23	14	127
St Epain	25		221		216	437
St Gilles	37	325	401	2	255	983
St Laurent d'Agny	37	67	236	53	9	365
Torreilles	20	128	128	85	121	462
Toulenne	191	4218	3518	3603	3476	14815
Ullensvang	10	231	29	23		283
Vienna	8	153	147	146	42	488

**Table 2:** Stages recorded in the experimental sites, when available, associated with the corresponding BBCH stage.

**Country**	**Location**	**Beginning of flowering**		**Full flowering**		**End of flowering**		**Maturity**	
Belgium	Gembloux	10% open flowers	*BBCH 61*	50% open flowers	*BBCH 65*	petal fall	*BBCH 67*	ripe fruits, harvest	*BBCH 89*
Bulgaria	Plovdiv	5% open flowers	*BBCH 61*	25–75% open flowers	*BBCH 65*	Petal falls for 75% of flowers	*BBCH 67*		
France	All experimental sites	10% open flowers	*BBCH 61*	80% open flowers	*BBCH 65*	10–20% petal fall	*BBCH 67*	30% ripe fruits (color, taste)	*BBCH 89*
Germany	Bonn	5–10% open flowers	*BBCH 61*	50–75% open flowers to beginning of petal fall	*BBCH 65*		*BBCH 67*		
Germany	Jork	1% open flowers	*BBCH 60*	80% open flowers	*BBCH 65*	Petal falls for 90% of flowers	*BBCH 67*		
Italy	Forli	5–10% open flowers	*BBCH 61*	80% open flowers	*BBCH 65*	No more pollen is shed by the open flowers	*BBCH 67*		
Norway	Ullensvang	1% open flowers	*BBCH 60*		*BBCH 65*		*BBCH 67*		
Serbia	Čačak	10–20% open flowers	*BBCH 61*	90–100% open flowers	*BBCH 65*	90% petal fall	*BBCH 67*		
Spain	Cieza	5–10% open flowers	*BBCH 61*	50–75% open flowers to beginning of petal fall	*BBCH 65*	75% open flower to the most petals have fallen	*BBCH 67*		
Spain	Jumilla	5–10% open flowers	*BBCH 61*	50–75% open flowers to beginning of petal fall	*BBCH 65*	75% open flower to the most petals have fallen	*BBCH 67*		
Spain	Lleida	1% open flowers	*BBCH 60*	80% open flowers	*BBCH 65*	100% open flowers	*BBCH 67*		
Switzerland	Changins			80% open flowers	*BBCH 65*				
United Kingdom	East Malling	1% open flowers	*BBCH 60*	50% open flowers	*BBCH 65*	90% petal fall	*BBCH 67*		

**Table 3:** Metadata.

**Header**	**Description**
Country	
Institute	Research or experimental institute attached to the experimental station
Station	Name of station
Latitude	Latitude in decimal degrees
Longitude	Longitude in decimal degrees
Altitude	Altitude in meters
Plantation	Year of plantation of the tree
Year	Year of observation
Cultivar	Registered name of the cultivar
Clone	Number of clone
Rootstock	Name of the rootstock
Beginning of flowering (date)	Date observed for the BBCH stage corresponding to beginning of flowering
Full flowering (date)	Date observed for the BBCH stage corresponding to full flowering
End of flowering (date)	Date observed for the BBCH stage corresponding to end of flowering
Beginning of maturity (date)	Date observed for the BBCH stage corresponding to maturity
Beginning of flowering	Number of days in year [1–365/366] for the BBCH stage corresponding to beginning of flowering
Full flowering	Number of days in year [1–365/366] for the BBCH stage corresponding to full flowering
End of flowering	Number of days in year [1–365/366] for the BBCH stage corresponding to end of flowering
Beginning of maturity	Number of days in year [1–365/366] for the BBCH stage corresponding to maturity
Flowering duration	Number of days between beginning and end of flowering

**Table 4:** Consistency of data between sites.

**Compared sites**	**Distance (km)**	**Cultivar**	**Number of common years**	**Spearman correlation**	**Average date for beginning of flowering**	
Balandran	Etoile	123	Burlat	22	0.93	89
Balandran	Etoile	123	Ferdouce	15	0.67	82
Balandran	Etoile	123	Summit	26	0.84	93
Balandran	Etoile	123	Sweetheart Sumtare	21	0.87	85
Gembloux	Bonn	164	Burlat	20	0.85	104
Balandran	Toulenne	390	Arcina Fercer	18	0.95	87
Balandran	Toulenne	390	Burlat	24	0.88	87
Balandran	Toulenne	390	Coralise Gardel	15	0.57	81
Balandran	Toulenne	390	Duroni 3	15	0.77	94
Balandran	Toulenne	390	Earlise Rivedel	19	0.76	81
Balandran	Toulenne	390	Ferdouce	17	0.88	80
Balandran	Toulenne	390	Ferobri	18	0.84	85
Balandran	Toulenne	390	Fertard	16	0.68	92
Balandran	Toulenne	390	Folfer	16	0.93	81
Balandran	Toulenne	390	Hedelfingen	21	0.88	90
Balandran	Toulenne	390	Kordia	17	0.72	88
Balandran	Toulenne	390	Lapins	20	0.78	84
Balandran	Toulenne	390	Napoleon	18	0.90	87
Balandran	Toulenne	390	Rainier	27	0.88	85
Balandran	Toulenne	390	Regina	17	0.79	92
Balandran	Toulenne	390	Stark Hardy Giant	24	0.90	85
Balandran	Toulenne	390	Summit	29	0.84	93
Balandran	Toulenne	390	Sweetheart Sumtare	19	0.87	85
Balandran	Toulenne	390	Van	17	0.90	83
Toulenne	Etoile	411	Burlat	28	0.93	89
Toulenne	Etoile	411	Duroni 3	16	0.87	96
Toulenne	Etoile	411	Summit	26	0.91	93
Toulenne	Etoile	411	Sweetheart Sumtare	15	0.86	85
Spearman correlation and average date for beginning of flowering (BBCH 61) were calculated for cultivars grown in the different sites.						
